# 3D Internal Visualization of Concrete Structure Using Multifaceted Data for Ultrasonic Array Pulse-Echo Tomography

**DOI:** 10.3390/s21196681

**Published:** 2021-10-08

**Authors:** Hungjoo Kwon, Changbin Joh, Won Jong Chin

**Affiliations:** Korea Institute of Civil Engineering and Building Technology, Gyeonggi, Goyang 10223, Korea; cjoh@kict.re.kr (C.J.); wjchin@kict.re.kr (W.J.C.)

**Keywords:** 3D internal visualization, non-destructive evaluation, ultrasonic array pulse-echo tomography, synthetic aperture focusing technique, concrete structure

## Abstract

This research proposes a 3D internal visualization using ultrasonic pulse-echo tomography technique to evaluate accurately the state of concrete structures for their efficient maintenance within a limited budget. Synthetic aperture focusing technique (SAFT) is used as a post-processing algorithm to manipulate the data measured by the ultrasonic pulse-echo technique. Multifaceted measurements improve the weakness of the existing ultrasonic pulse-echo tomography technique that cannot identify the area beyond a reflector as well as the area located far away from measuring surfaces. The application of apodization factor, pulse peak delay calibration and elimination of trivial response not only complements the weaknesses of the SAFT algorithm but also improves the accuracy of the SAFT algorithm. The results show that the proposed method reduces the unnecessary surface noise and improves the expressiveness of the reflector’s boundaries on the resulting images. It is expected that the proposed 3D internal visualization technique will provide a useful non-destructive evaluation tool in combination with another structure evaluation method.

## 1. Introduction

Despite the crucial role played by infrastructure in providing the nation’s essential services and maintaining its economic activities [1], the American Society of Civil Engineers gave a D+ grade to the infrastructure of the US in 2017 and estimated the cost for its improvement to USD 4.5 trillion by 2026 [2]. To worsen the situation, the estimated cost necessary to repair the infrastructure escalates gradually every year [3]. Therefore, there is an urgent need for innovative technologies and processes to inspect and evaluate the conditions of the infrastructure more effectively and efficiently with less monetary resources [4].

Ultrasonic pulse-echo tomography is a non-destructive structural inspection technique that makes it possible to evaluate and visualize the inside of concrete structures precisely [5,6,7]. A1040 MIRA is one of the popular ultrasonic pulse-echo tomography devices providing a sectional image of concrete structures [8,9,10]. The MIRA device consists of an array of ultrasonic transducers, which measure multiple ultrasonic pulse-echo signals with a single scan and calculate a cross-sectional image of a target using an embedded image reconstruction algorithm called to SAFT-C algorithm [11]. The SAFT-C algorithm is a time-domain SAFT algorithm based on the delay-and-sum method that focuses reflections in accordance with delayed times [12]. Although a frequency-domain SAFT algorithm, such as the wavenumber [13] and migration algorithm [14], which manipulate the Fast Fourier Transform to calculate resulting images, show a faster calculation speed and better resolution [15], the time-domain SAFT algorithm seemed to be adapted in the MIRA device due to its simplicity and low-performance requirement on a microprocessor [16].

In addition, a 3D volumetric image can be obtained with a software program, IntroView Concrete, supported by the MIRA’s manufacturer [17,18]. IntroView Concrete is known to construct a general 3D volumetric image for NDE (Non-Destructive Evaluation) by interpolating 2D sectional images [19,20,21,22]. Although this interpolated image allows an intuitive and immediate visualization of the inside of concrete structures, the information at the interpolated area becomes unreliable as the spacing between 2D images increases. Also, a reflector arranged in a specific direction is highly improbable because the MIRA device uses a polarized shear wave [18,23] and the MIRA’s software cannot combine data measured in multiple orientations to create an image. Furthermore, the detectability at the area beyond a reflector is low as a result of the high reflectivity of the shear wave [18].

In this research, a new method to create a volumetric image made of multifaceted signal data is proposed to improve the quality of the resulting image by the ultrasonic pulse-echo tomography. Utilizing multifaceted signals allows representation of the reflectors inside more precisely than single faceted signals like the MIRA. The proposed algorithm is based on the SAFT (Synthetic Aperture Focusing Technique) algorithm [24,25,26,27] that can combine data measured in multiple directions to compute the resulting images. A beam pattern of the transducer in the MIRA device is considered by applying a weighting factor called the apodization factor [28,29,30]. A reflection sagging as a result of long ultrasonic pulse duration is complemented by the pulse peak delay calculation [31]. In the last step, trivial reflections like back-wall reflections are eliminated to emphasize the internal reflections.

## 2. Experiment

### 2.1. Concrete Cube Specimen

**Figure 1:**
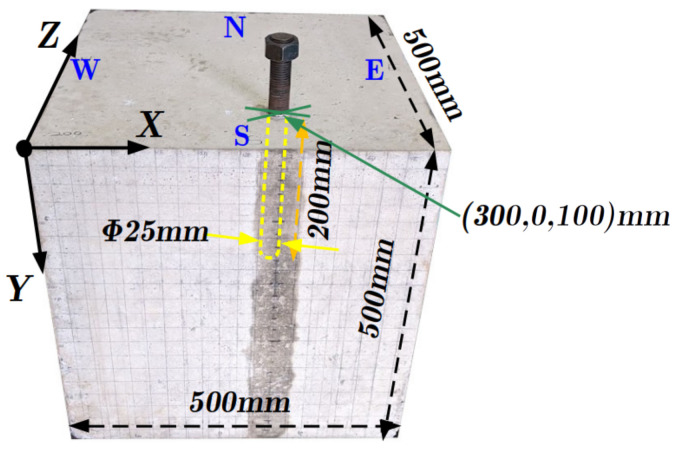
Plain concrete cube specimen with steel rod.

The plain concrete cube (500 × 500 × 500 mm) shown in Figure 1 and made of normal concrete with a strength of 27 MPa and small-sized aggregates to avoid wave scattering by the aggregates is adopted for the experiment. A steel rod with a diameter of 25 mm is inserted vertically in the concrete specimen at (x, y, z) = (300, 0, 100) mm to a depth of 200 mm to mimic damage or a reflector. The lateral surfaces are indicated by S, N, W, and E with respect to their direction. An ultrasonic speed of 2300 m/s in the concrete specimen was measured on average by the MIRA device.

### 2.2. Ultrasonic Array System

**Figure 2:**
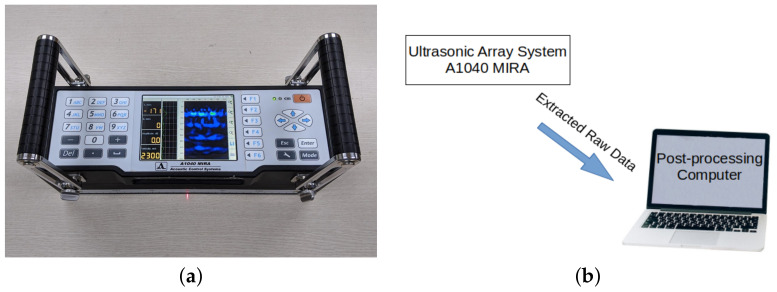
(**a**) Ultrasonic array system, A1040 MIRA and (**b**) experiment setup.

The ultrasonic array device employed in this research is A1040 MIRA manufactured by Acoustic Control System as shown in Figure 2a. The device comprises 4 × 12 transducers, and each 4-transducers in a column acts as one transducer. Thus, the device operates as a 12-ultrasonic-transducer array. The MIRA device enables the measurement of multiple time-series signals along multiple paths at multiple locations with a single scan. A time-series signal for a single transducer pair is called A-scan, and the device generates 66 A-scans for all possible transducer pairs except for identical transducers with a single scan within 2 to 3 s. This scanning scheme is called full matrix capture. The full matrix capture enables the enlargement of the inspecting area and the increased resolution of the resulting images [32]. The measured A-scan data is directly extracted from the MIRA and post-processed in a computer to calculate the resulting images as shown in Figure 2b.

An ultrasonic transducer in the array system must generate a widespread beam pattern so that the furthest transducer pair in the array device receives a distinguishable ultrasonic signal among the noise. In order for the transducer to have the widespread beam pattern, the diameter of the transducer should be as small as a point. This point-like transducer called DPC (Dry Point Contact) transducer does not require a couplant [33,34]. The MIRA device, therefore, can scan quickly without applying the couplant to scan surfaces every time.

The shear wave DPC transducer in the MIRA device shakes its tip perpendicularly to the direction of wave propagation [35] to create an anisotropic beam pattern. This beam pattern is compensated in the SAFT algorithm. The polarization of the shear wave lowers the detectability of a reflector perpendicular to the direction of wave and polarization. To solve this issue, a target is usually scanned horizontally as well as vertically to identify both directions of reflectors.

### 2.3. Measurement

**Figure 3:**
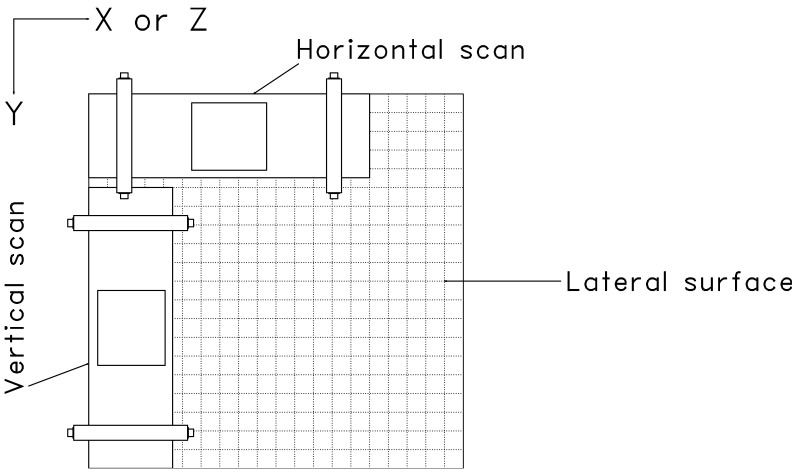
Scan orientation of MIRA device.

To evaluate the effect of the number of scanned surfaces on the resulting images, all four lateral surfaces (S, N, W, and E) are scanned. A grid with spacing of 25 mm is drawn on all lateral surfaces. Considering that the central frequency of a shear wave DPC transducer in the MIRA device is 50 kHz, the frequency of the ultrasonic pulse is set to 50 kHz to maximize the performance of the MIRA device. Each surface is additionally scanned vertically to increase the detectability of reflectors [18] as shown in Figure 3. Raw signal data is extracted from the MIRA device.

## 3. 3D SAFT Algorithm

The raw signal data measured by the MIRA device can rarely be interpreted intuitively. Thus, an image reconstruction algorithm is required to transform the measured signal data into an understandable image. The 3D SAFT algorithm based on the delay-and-sum method is used to transform the measured signal into a volumetric image.

**Figure 4:**
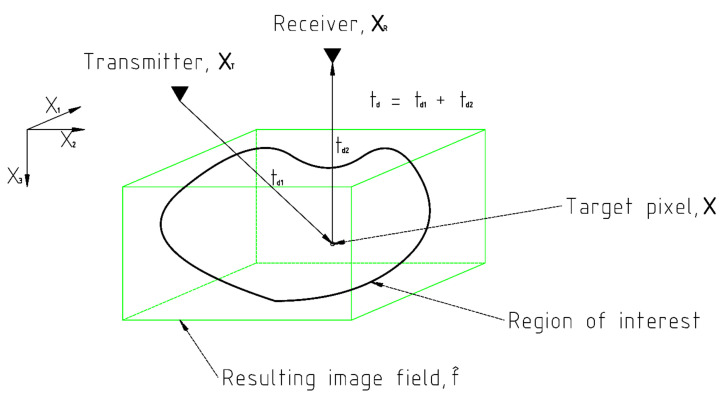
Geometry of SAFT algorithm.

The geometry of the 3D SAFT algorithm is plotted in Figure 4. $f(x_{1},x_{2},x_{3})$ is the unknown status of the region of interest (ROI) that the SAFT algorithm is about to figure out. The measured signal *e* is as follows.

(1) $$e\left(t,x_{T},x_{R}\right)=\overset{\infty }{\underset{-\infty }{\;\int \int \int \;}}f\left(x_{1},x_{2},x_{3}\right)\delta \left(t-t_{d}\right)dx_{1}dx_{2}dx_{3}$$

where *t* is the time; $x_{T}$ and $x_{R}$ are the transmitting and receiving transducer, respectively; δ is the Dirac delta function; $x_{1}$, $x_{2}$, and $x_{3}$ are the Cartesian coordinates; and $t_{d}$ is the delayed time as follows.

(2) $$t_{d}=\frac{\sqrt{{\left(x_{1}-x_{T_{1}}\right)}^{2}+{\left(x_{2}-x_{T_{2}}\right)}^{2}+{\left(x_{3}\right)}^{2}}+\sqrt{{\left(x_{1}-x_{R_{1}}\right)}^{2}+{\left(x_{2}-x_{R_{2}}\right)}^{2}+{\left(x_{3}\right)}^{2}}}{c}$$

where *c* is the wave speed.

The unknown wave field of ROI is estimated by superimposing the amplitude values of A-scans at the corresponding delayed times. The estimated field $\hat{f}$ is given by

(3) $$\hat{f}\left(x_{1},x_{2},x_{3}\right)=\sum_{x_{T}=1}^{N_{e}-1}\sum_{x_{R}=X_{T}+1}^{N_{e}}e\left(t,x_{T},x_{R}\right)$$

The estimated wave field of ROI has data with a sinusoidal form. In order to identify the intensity at each point in the ROI, an envelope curve of the estimated wave field is calculated by the Hilbert transform. Then, the field is normalized to the maximum value.

The computation speed of the algorithm is heavily dependent on the size of the resulting image [36]. In order for the MIRA device to be used for in-situ inspection, the size of the resulting image seems to be limited to 432 by 269 pixels allowing it to be complete within 2 to 3 s. The dimension of the proposed 3D algorithm’s output is selected as 51 by 51 by 51 pixels to match up with the MIRA’s output.

### 3.1. Results

**Figure 5:**
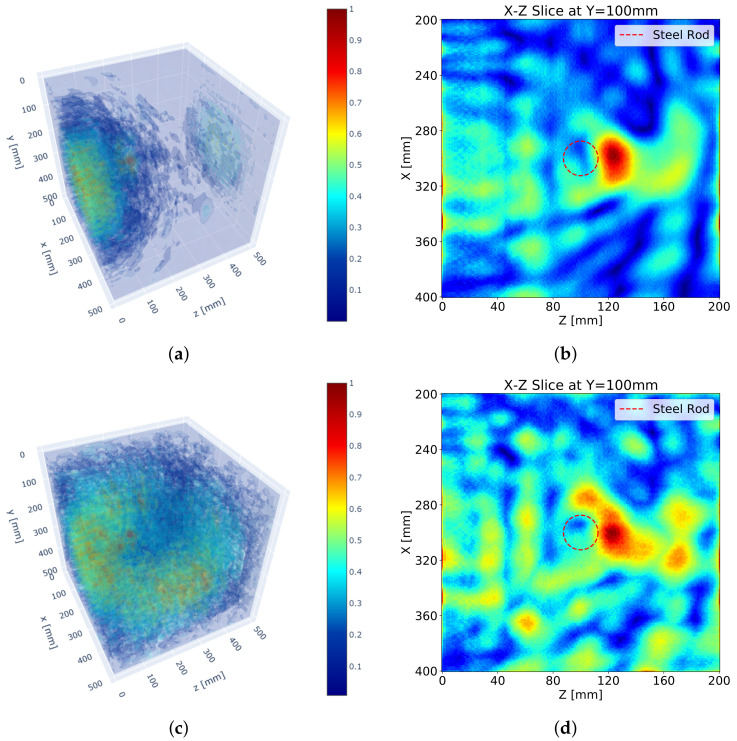
SAFT results: (**a**) 3D visualization with S surface measurement, (**b**) sectional visualization at *y* = 100 mm with S surface measurement, (**c**) 3D visualization with S, N, W, and E surface measurements, (**d**) sectional image at *y* = 100 mm with S, N, W and E surface measurement.

Figure 5 depicts the results of the 3D SAFT algorithm. Figure 5a,b show the 3D visualization resulting from the S surface measurement only, and Figure 5c,d represent the visualization resulting from the measurements of all lateral surfaces. A reflection for the steel rod is slightly identifiable, but an unintended reflection noise near the measuring surface is formed. The surface noise expanded by the increased number of surface measurements even hinders the detection of the reflection for the steel rod. This phenomenon results from the ultrasonic DPC transducer that transmits a surface wave as well as a shear wave. The surface wave is a wave that travels along the shortest path, surface, from a transmitter to a receiver. Thus, the surface wave usually appears at the very first part of the measured received signal data. This surface wave impairs the beginning of the measured signal and makes it difficult to distinguish a reflected wave signal from it. Besides, a strong magnitude of the surface wave makes it difficult to identify reflectors.

In addition, the reflection is not formed at the exact location, but at the boundary of the steel rod near the measuring surface as shown in Figure 5b. The reflection is developed beyond the reflected point. This phenomenon results from a long pulse duration of the ultrasonic pulse generated by the DPC transducer. Each ultrasonic transducer has its own frequency bandwidth which makes an output ultrasonic pulse longer than an input electronic signal by eliminating high-frequency components. As a result, the reflection for a reflector becomes long and ragged and the peak of the reflection sags further against the measuring surface proportional to the length of the pulse.

An increased number of measuring surfaces does not improve the performance of the SAFT algorithm as shown in Figure 5d. Although the reflections for the steel rod from all surface measurements are included, the unfocused reflections interrupt the detection of the steel rod. In addition, the increased surface noise and the back-wall reflection from the N surface measurement interfere with the detection of the steel rod as well.

### 3.2. Apodization Factor

**Figure 6:**
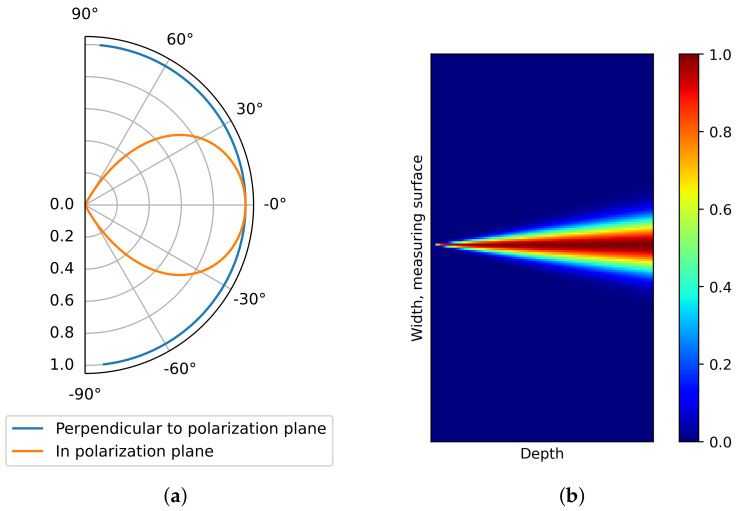
(**a**) Ultrasonic DPC transducer beam pattern and (**b**) apodization factor field for polarization plane beam pattern.

The disclosed beam pattern of the shear wave DPC transducer [37] is simulated in Figure 6a. The beam pattern in the polarization plane is anisotropic while the beam pattern perpendicular to the polarization plane is isotropic. As the 3D SAFT algorithm mentioned above did not consider the beam pattern, unnecessary noises are dispersed in the resulting images.

The weighting factor considering the beam pattern in the SAFT algorithm is called the apodization factor, *A*. The SAFT equation modified for the beam pattern is given by

(4) $$\hat{f}\left(x_{1},x_{2},x_{3}\right)=\sum_{x_{T}=1}^{N_{e}-1}\sum_{x_{R}=X_{T}+1}^{N_{e}}A\times e\left(t,x_{T},x_{R}\right)$$

In this research, a Hann window is used for the apodization factor. The equation of the apodization factor is as follows.

(5) $$A=\alpha \left(x,z,x_{T}\right)\times \alpha \left(x,z,x_{R}\right)$$

(6) $$\alpha \left(x,z,x^{*}\right)=\left\{\begin{array}{cc} \frac{1}{2}\left(1+\cos\left(2\pi \hat{x}\right)\right) & |\hat{x}|<\frac{1}{2}\\ 0 & \mathrm{otherwise} \end{array}\right.$$

where *x* and *z* are pixel positions in width and depth, respectively, $x^{*}$ is position of transducer in width, $\hat{x}=\left(x-x^{*}\right)/\Delta x\left(z\right)$ is normalized coordinate in width, Δx(z)=2ztan(Δθ/2), and Δθ is the angular beam width of the transducer which is $130^{\circ }$. Figure 6b illustrates the apodization factor.

**Figure 7:**
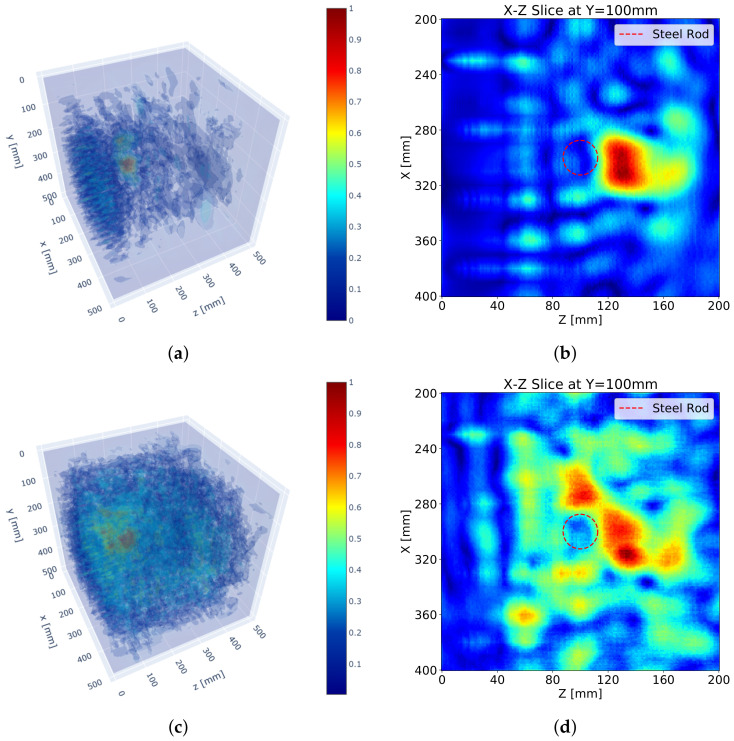
SAFT results with apodization factor: (**a**) 3D visualization with S surface measurement, (**b**) sectional visualization at *y* = 100 mm with S surface measurement, (**c**) 3D visualization with S, N, W, and E surface measurements and (**d**) sectional visualization at *y* = 100 mm with S, N, W, and E surface measurements.

Figure 7 describes the SAFT results after the implementation of the apodization factor. Compared with the SAFT results without the apodization factor in Figure 5, the noise near the measuring surface is significantly erased. As a result, a clearer discrimination of the reflection for the steel rod can be performed. This means the location of the reflection can be recognized in the volumetric image.

However, the noise far from the measuring surface is still considerable and obstructs the detection of reflectors. Also, the low accuracy of the SAFT algorithm as shown in Figure 7b,d lowers the detectability of the algorithm. The increased number of the measuring surfaces enhances the reflections for the steel rod but also enlarges the noise. Although the existence of the steel rod is identifiable, the shape of the steel rod is not identifiable.

### 3.3. Pulse Peak Delay

**Figure 8:**
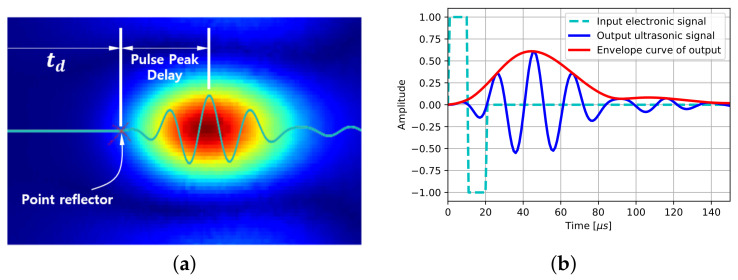
(**a**) Pulse peak delay on SAFT result and (**b**) input and output signal of DPC transducer.

The delayed time $t_{d}$ in the SAFT algorithm is the time between the beginning of the signal and of the pulse if a point reflector is assumed as shown in Figure 8a. The size of a reflection depends on the duration of the pulse. Although the point reflector is located at the beginning of the pulse, the peak of the reflection is placed beyond the reflector as much as the pulse peak delay. A short pulse can ignore the pulse peak delay in the SAFT algorithm, but the DPC transducer generating a long pulse has to take into account the pulse peak delay to improve the quality of the algorithm.

An ultrasonic transducer serves as a signal filter to an input signal. The DPC transducer has a central frequency of 50 kHz and a frequency bandwidth of 20 kHz. A one-cycle square input signal of the DPC transducer is transformed into a sinusoidal long pulse by eliminating low- and high-frequency components as shown in Figure 8b.

The peak of the output signal is located at about 45 μs, and this amount is reflected in the SAFT results. The slack length in this media where the wave speed is 2300 m/s is 103.5 mm, and the slack distance in the SAFT results is half the length due to the pulse-echo relationship. This leads the strongest reflection point to be placed about 50 mm beyond the exact location in Figure 7b.

A modified delayed time considering the pulse peak delay is the sum of the delayed time $t_{d}$ and the pulse peak delay. This modified delayed time term substitutes for the delayed time term in the SAFT algorithm to consider the pulse peak delay and improve the quality of the resulting images.

**Figure 9:**
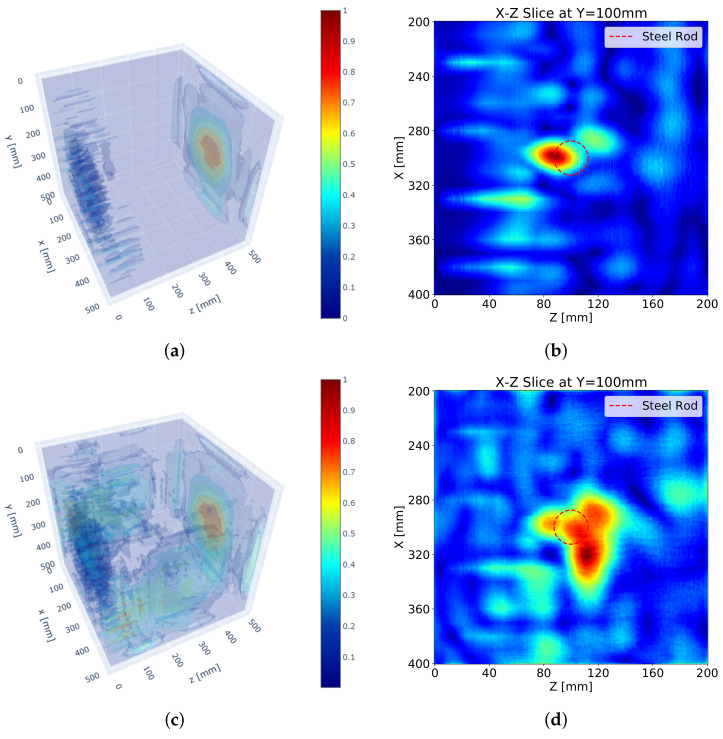
SAFT results with pulse peak delay and apodization factor: (**a**) 3D visualization with S surface measurement, (**b**) sectional visualization at *y* = 100 mm with S surface measurement, (**c**) 3D visualization with S, N, W, and E surface measurements and (**d**) sectional visualization at *y* = 100 mm with S, N, W, and E surface measurements.

Figure 9 shows the SAFT results after the application of the pulse peak delay as well as the apodization factor. First of all, the accuracy of the SAFT algorithm improved, and the noise around the wave field was reduced in general. It seems that the improved accuracy of the algorithm decreases the noise by defocusing. Additionally, the high accuracy intensified the back-wall reflection. As a result, the reflection for the steel rod was relatively diminished.

Figure 9b,d show that the reflections are developed at the exact reflecting location, that is the boundary of the steel rod. Especially, the peak of the reflection by the S surface measurement is formed exactly on the boundary. The other reflections by the measurements on the other surfaces have relatively lower accuracies due to far distances, but the total reflections are enough to display the shape of the reflector.

## 4. Discussion

**Figure 10:**
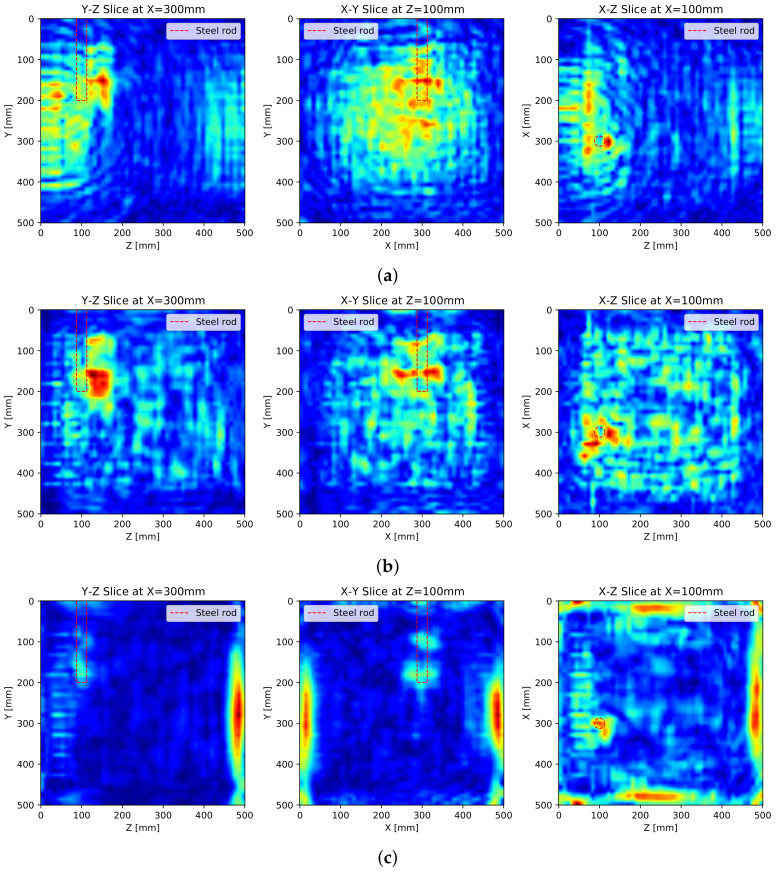
Cross-sectional images of SAFT results with all surfaces measurements: (**a**) basic SAFT, (**b**) SAFT with apodization factor and (**c**) SAFT with apodization factor and pulse peak delay calibration.

For detailed analysis, the cross-sectional images of the SAFT results with all surface-measurements are illustrated in Figure 10 where Figure 10a are the results of basic SAFT, Figure 10b are the SAFT results after the apodization factor, and Figure 10c are the SAFT results after the apodization factor and the pulse peak delay calibration, respectively. Each sectional image represents an axial image centered at the middle of the steel rod.

The noise formed in the SAFT results can be roughly categorized into two groups: surface noise by a surface wave and internal noise by defocusing. The apodization factor dealing with the beam pattern of the DPC transducer removes the noise near the measuring surface significantly, and the pulse peak delay calibration reduces internal noise by defocusing through the increment of accuracy.

The pulse peak delay calibration not only reduces the internal noise but also improves the performance of the SAFT algorithm. After applying the pulse peak delay calibration, the reflections are formed at the exact reflecting point, and the back-wall reflections appear on the resulting images.

In Figure 10c, the back-wall reflections have the strongest reflections in the images as a significant portion of the ultrasonic pulse is reflected at the back wall. The reflection for the steel rod also seems to be weakened by the intense back-wall reflection. Although this phenomenon is normal, the weakened reflection can disturb the interpretation of the 3D internally visualized image.

**Figure 11:**
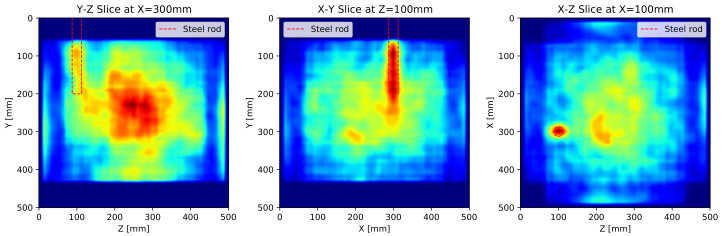
Cross-sectional images of SAFT results with all measurements by MIRA.

The cross-sectional images of SAFT by the MIRA device are represented in Figure 11. Since the software provided by the MIRA manufacturer does not allow to combine multifaceted measurements, the presented cross-sectional images are calculated by superposition and interpolation of the results by the MIRA device. The area near *y* = 0 and 500 mm is not evaluated as the MIRA results do not cover that area.

In the cross-section of the X-Z plane in Figure 11, the reflection of the steel rod is strong enough to be distinguished from the noise. However, the reflection of the steel rod is longer than the actual length in the cross-section of the Y-Z and X-Y planes. It is perceived that the MIRA algorithm manipulates signals from all angles in contrast to the apodization factor weakening signal from the side lobe.

The reflection of the steel rod in the X-Z plane is recognized as a single reflection, even though all lateral measurements are combined, and the reflection is formed at the center of the steel rod and not at the boundary. It can be observed that the MIRA’s algorithm does not focus the reflection exactly and this defocusing results in the noise at the center of the concrete cube specimen.

**Figure 12:**
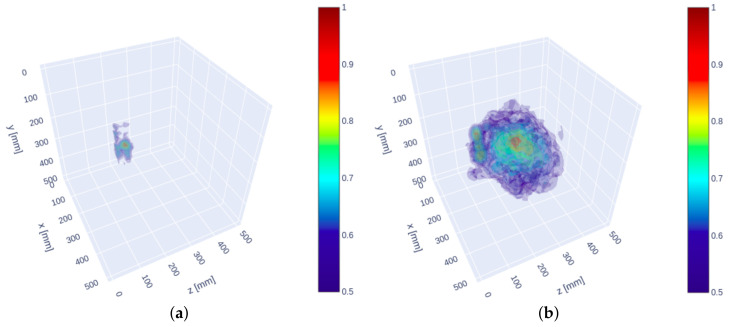
3D visualization of SAFT results (**a**) by the proposed algorithm and (**b**) by the MIRA results.

Figure 12 presents the 3D visualization (a) of the proposed SAFT results and (b) of the MIRA results. Trivial reflections like surface wave and back-wall reflections were eliminated from these 3D visualizations. The clear reflection of the internal reflector in Figure 12a only enables intuitively to interpret the result as compared to the MIRA results. On the other hand, the MIRA results represent the shape of the steel rod explicitly, but the strong noise at the center by the defocusing prevents the recognition of the actual internal shape of the specimen.

## 5. Conclusions

A new 3D internal visualization technique modified from the basic SAFT algorithm by using the ultrasonic pulse-echo tomography was proposed to allow an intuitive structural evaluation of concrete structures. The proposed technique including the apodization factor and the pulse peak delay calibration improved the quality of the resulting images and weakened the noise in the results. Also, the manipulation of all possible surface measurements improved the quality of the resulting images. In addition, the intuitive interpretation of the resulting images could be increased through the elimination of definite reflections like back-wall and surface noise reflections.

The final processed image minimizing the unnecessary reflections made it possible to visualize clearly the internal reflectors. However, the results showed that the resolution of the resulting images needed improvement to quantify the internal reflectors. The quantification of the resulting images requires high-resolution images and the high-resolution images require a transducer transmitting a short pulse. Accordingly, an accurate interpretation of the results necessitates the development of hardware like a transducer as well as software such as image reconstruction algorithms.

The proposed 3D internal visualization SAFT algorithm provided more accurate results than the 2D SAFT algorithm interpolating 2D images to create a 3D image. However, the proposed algorithm requires significant computational loads proportional to the size of the resulting image. The proposed 3D visualization methodology will provide great diagnostic power onsite with the development of a faster microprocessor and larger storage in a portable device.

## Data Availability

Data sharing not applicable.
